# CHST12: a potential prognostic biomarker related to the immunotherapy response in pancreatic adenocarcinoma

**DOI:** 10.3389/fendo.2023.1226547

**Published:** 2024-01-25

**Authors:** Kun Liu, Lu Li, Guang Han

**Affiliations:** Department of Anesthesiology, Shengjing Hospital of China Medical University, Shenyang, Liaoning, China

**Keywords:** immune cell, infiltration, glycosylation (enzymatic conjugation with carbohydrates), prognostic signature, pancreatic adenocarcinoma (PAAD), tumor microenvironment

## Abstract

**Background:**

Pancreatic adenocarcinoma (PAAD) is characterized by lower immunogenicity with a poor response rate to immune checkpoint inhibitors (ICIs) and exhibits the poorest prognosis of all solid tumors, which results in the highest tumor-related mortality among malignancies. However, the underlying mechanisms are poorly understood. In addition, diverse carbohydrate sulfotransferases (CHSTs), which are involved in the sulfation process of these structures, play an important role in the metastatic spread of tumor cells. Aberrant glycosylation is beginning to emerge as an influencing factor in tumor immunity and immunotherapy. Therefore, it might serve as a biomarker of the immunotherapeutic response in tumors. The purpose of the study was to evaluate the role of CHST12 in PAAD prognosis and its relevance to the immunotherapeutic response.

**Methods:**

A comprehensive investigation of the interactions between CHST12 expression and the immune microenvironment as well as the clinical significance of CHST12 in PAAD was conducted. Data derived from the Cancer Genome Atlas (TCGA) database were analyzed using univariate and multivariate approaches, the Tumor Immune Estimation Resource (TIMER), and Tumor Immune Dysfunction and Exclusion (TIDE) algorithms. Publicly available datasets were analyzed in this study. These data can be found on websites such as http://www.xiantao.love and https://www.proteinatlas.org. An assessment of the predictive value of CHST12 for PAAD prognosis was conducted using univariate and multivariate Cox regression analysis, Kaplan–Meier analysis, and nomograms. The TIMER algorithm calculates the proportions of six types of immune cells. The TIDE algorithm was used to indicate the characteristics of tumors that respond to ICI therapy.

**Results:**

The mRNA and protein levels of CHST12 showed the opposite trend. CHST12 mRNA expression was significantly upregulated in PAAD. According to Cox regression analysis, CHST12 RNA expression acts as a protective factor for overall survival [hazard ratio (HR), 0.617, P < 0.04]. Functional annotation indicated that CHST12-associated differentially expressed genes (DEGs) were related to the signaling activity of receptor tyrosine kinases and the regulation of ubiquitin-protein transferase. These are usually involved in tumor development and may be related to the treatment responses of immune checkpoint inhibitors (ICIs). There was significantly higher CHST12 mRNA expression in PAAD samples than in non-malignant samples.

**Conclusions:**

In PAAD, elevated CHST12 mRNA expression might regulate immune cell infiltration into the tumor microenvironment (TME) and may predict clinical outcomes.

## 1 Introduction

Pancreatic adenocarcinoma, commonly referred to as pancreatic cancer, is a type of cancer that begins in the pancreas, an organ located deep within the abdomen (1). The pancreas plays a crucial role in digestion and blood sugar regulation by producing enzymes and hormones, such as insulin and glucagon (2, 3). PAAD is a highly aggressive malignancy, exhibiting the poorest clinical outcome of all solid tumors. Generally, when patients come to the hospital, they are diagnosed with advanced PAAD. It is characterized by lower immunogenicity, with a lower number of predicted neoantigens, low immune infiltration, and poor response rates to ICI therapy, with a median survival of 6 months and growing incidence rates worldwide (4–7). The low tumor-infiltrating immune cell microenvironment features of PAAD consist of cancer cells, fibroblasts, myofibroblasts, endothelial cells, tumor-infiltrating lymphocytes (TILs), and extracellular matrix.

The majority of pancreatic cancers are adenocarcinomas, which start in the cells that line the ducts of the pancreas (8, 9). There are other less common types of pancreatic cancer, such as neuroendocrine tumors and acinar cell carcinomas, leading to drug resistance and great inertia in the response rates to immunotherapy (10, 11). Therefore, it is particularly difficult to treat PAAD. Specific targeted therapies such as EGFR-targeted therapy confer a significant survival benefit in a minority of PAAD patients who are wild-type KRAS patients (12, 13). Approximately 90% of pancreatic cancers harbor oncogenic KRAS mutations (14, 15). With the improvement in many advanced medical diagnostic technologies, such as endoscopic ultrasound, the early diagnosis rate of PAAD has been greatly improved (16, 17). However, given the limited effectiveness of current treatments for PAAD, new promising biomarkers are urgently needed to improve the prognosis and treatment of PAAD.

As a fundamental and prominent post-translational modification of proteins, the involvement of glycosylation in cellular functions includes transformation, adhesion, cell growth, differentiation, and tumor immune surveillance (18, 19). Abnormal glycosylation has been implicated as a crucial mechanism that triggers tumor progression (20, 21). Dysregulation in glycosylation can affect the function, sensitivity and expression of cell-surface receptors. These features make glycosylation a critical molecular event in cancer pathology (22). Glycoproteins are currently the most commonly used clinical tumor biomarkers (e.g., the AFP test for liver cancer, CA125 test for ovarian cancer, CEA, CA199 test for colon cancer, and PSA test for prostate cancer) (23, 24). Subsequently, the role of aberrant glycosylation influences tumorigenesis, which affects growth, immune surveillance, and immunotherapy.

Many recent publications have indicated that glycosylation can lead to an improvement in the immunotherapeutic effect and confer better treatment outcomes in cancer (25–27). As such, further elucidating the molecular mechanisms and consequently promoting cancer immune evasion is urgently required (28). As one of the important components of the tumor microenvironment, proteoglycans promote the progression of cancers, such as lung cancer, pancreatic cancer, and GBM (29–31). Multiple family members are implicated in tumor occurrence and development (32). The current evidence suggests that carbohydrate sulfotransferase 12 (CHST12) is a cancer-related enzyme and is a potential biomarker in some tumor subtypes. For instance, GBMs with high CHST12 expression have a reduced survival rate and positive associations with KI67 expression (33). Qiang Ren et al. found that CHST12 was expressed at a significantly higher level in pancreatic tumors, highlighting the potential diagnostic utility of the CHST12 expression profile in pancreatic cancer (34). However, the specific functions and role of CHST12 in the prognosis of pancreatic cancer and the regulation of TME remain a mystery.

Here, we explored CHST12-related biological processes and CHST12-enhanced pathways to understand its functions in PAAD. The prognostic model based on univariate and multivariate Cox regression analysis revealed an independent prognosticator characteristic. Additionally, we used the TIDE algorithm to indicate the characteristics of CHST12 as a predictor for ICI therapy efficacy. The present study may help develop CHST12 as a prognostic biomarker and predict the efficacy of the immunotherapy treatment of PAAD patients.

## 2 Methods

### 2.1 Data processing

The level 3 HTSeq-FPKM (Fragments Per Kilobase per Million) sequencing RNA expression values were downloaded from the GTEx and TCGA datasets and log2-transformed [log2 (FPKM+1)]. The corresponding clinicopathological parameters were then obtained, including 171 normal tissue samples and 179 PAAD tumor samples. These databases are publicly open access. This study was approved by the Ethics Committee of the Shengjing Hospital of China Medical University (Ethical Approval Number: 2023PS700K).

### 2.2 Differentially expressed genes in PAAD

High-expression and low-expression groups of CHST12 were determined by the median expression from 178 PAAD patients. R (Version 3.6.3) was used to create plots with the ggplot2 package comparing the DEGs between the two groups using chi-square or Fisher exact tests (35). The following significance symbols were used: The ns value is equal to p≥0.05; * is equal to p< 0.05, ** is equal to p < 0.01, *** is equal to p < 0.001, and **** is equal to p < 0.0001.

### 2.3 Correlation analyses between CHST12 expression characteristics and the clinicopathology of PAAD

Detailed patient characteristics are shown in **Table 1**. Chi-square or Fisher exact tests were used to compare the clinicopathological characteristics of the 178 enrolled patients. Then, clinicopathological characteristics and CHST12 expression were analyzed using logistic regression.

**Table 1:** Clinicopathological characteristics of PAAD patients with differential CHST12 expression.

Characteristic	Low expression of CHST12	High expression of CHST12	p-value
n	89	89	
T stage, n (%)			0.810
T1	3 (1.7%)	4 (2.3%)	
T2	11 (6.2%)	13 (7.4%)	
T3	74 (42%)	68 (38.6%)	
T4	1 (0.6%)	2 (1.1%)	
N stage, n (%)			0.456
N0	23 (13.3%)	27 (15.6%)	
N1	66 (38.2%)	57 (32.9%)	
M stage, n (%)			1.000
M0	43 (51.2%)	36 (42.9%)	
M1	3 (3.6%)	2 (2.4%)	
Pathologic stage, n (%)			0.320
Stage I	7 (4%)	14 (8%)	
Stage II	77 (44%)	69 (39.4%)	
Stage III	1 (0.6%)	2 (1.1%)	
Stage IV	3 (1.7%)	2 (1.1%)	
Radiation therapy, n (%)			0.209
No	62 (38%)	56 (34.4%)	
Yes	18 (11%)	27 (16.6%)	
Primary therapy outcome, n (%)			0.208
PD	30 (21.6%)	19 (13.7%)	
SD	3 (2.2%)	6 (4.3%)	
PR	7 (5%)	3 (2.2%)	
CR	34 (24.5%)	37 (26.6%)	
Gender, n (%)			0.651
Female	42 (23.6%)	38 (21.3%)	
Male	47 (26.4%)	51 (28.7%)	
Age, mean ± SD	66.37 ± 10.58	63.12 ± 10.83	**0.045**

PAAD, pancreatic adenocarcinoma. Bold p-values represent significant relationships (p < 0.05).

### 2.4 Clinical significance of CHST12 expression in PAAD

Receiver operating characteristic (ROC) curve analysis was used to evaluate the diagnostic value of CHST12 for PAAD. Then, a prognostic analysis of CHST12 in PAAD patients was evaluated using the Kaplan–Meier method, univariate Cox proportional hazards regression, and multivariate Cox regression analysis, which were performed using the “survminer” and “survival” R packages. Published studies provide clinical outcomes data of PAAD patients, including OS, progression-free intervals (PFIs), and disease-specific survival (DDS). Analyses of statistical data were conducted using R (version 3.6.3). Significance values are defined as P-values less than 0.05.

### 2.5 Analysis of CHST12-associated differentially expressed genes in PAAD with functional annotations

DEGs identified between high and low expression groups of CHST12 mRNA were analyzed and processed through functional enrichment analysis with the Metascape database, Gene Ontology (GO) terms, and Kyoto Encyclopedia of Genes and Genomes (KEGG) pathways. In addition, reactome gene sets (https://metascape.org/gp/index.html#/main/step1) were included (36). The resultant terms were grouped into features based on attribute similarity, with a p-value threshold of 0.01 (< 0.01) and a minimum of three enriched genes.

### 2.6 Infiltration of immune cells into PAAD is associated with CHST12 expression

Tumor Immune Estimation Resource (TIMER) is an online database that uses a deconvolution statistical method, the single-sample GSEA (ssGSEA), to evaluate the TME immune cell infiltration levels from gene expression profiles (37–39). Via gene modules, TILs, including CD8^+^ T cells, B cells, CD4^+^ T cells, neutrophils, macrophages, and dendritic cells, were evaluated in PAAD in relation to CHST12 mRNA expression. The website (http://cis.hku.hk/TISIDB/) analyzes the relationship between tumors and the innate immune system (40). A database of tumor-immune system interactions called the Tumor-Immune System Interaction Database (TISIDB) was used to validate the relationship between CHST12 expression and TILs in PAAD using Pearson’s correlation analysis. Finally, a comparison of the distributions of immune checkpoint-associated genes (*SIGLEC15*, *IDO1*, *CD274*, *HAVCR2*, *PDCD1*, *CTLA4*, *LAG3*, and *PDCD1LG2*) was performed between the high and low expression groups of CHST12. Statistical significance was examined using the Wilcox test. The asterisks represent the degree of importance (*p < 0.05, **p < 0.01, ***p < 0.001).

### 2.7 Analysis of tumor immune dysfunction and exclusion

Using the gene exclusion model for tumor immune dysfunction, Tumor Immune Dysfunction and Exclusion (TIDE) model predicts cancer sensitivity to immune checkpoint therapy. The TIDE model includes dysfunctional T cells and the exclusion of T-cell mechanisms of immune evasion by tumors (41). The TIDE score indicates the potential for tumor immune escape. A high TIDE score suggests a bad curative effect of ICIs and a short survival time after ICI treatment in patients.

## 3 Results

### 3.1 Expression profiles of CHST12 and prognosis value in PAAD

**Figure 1:**
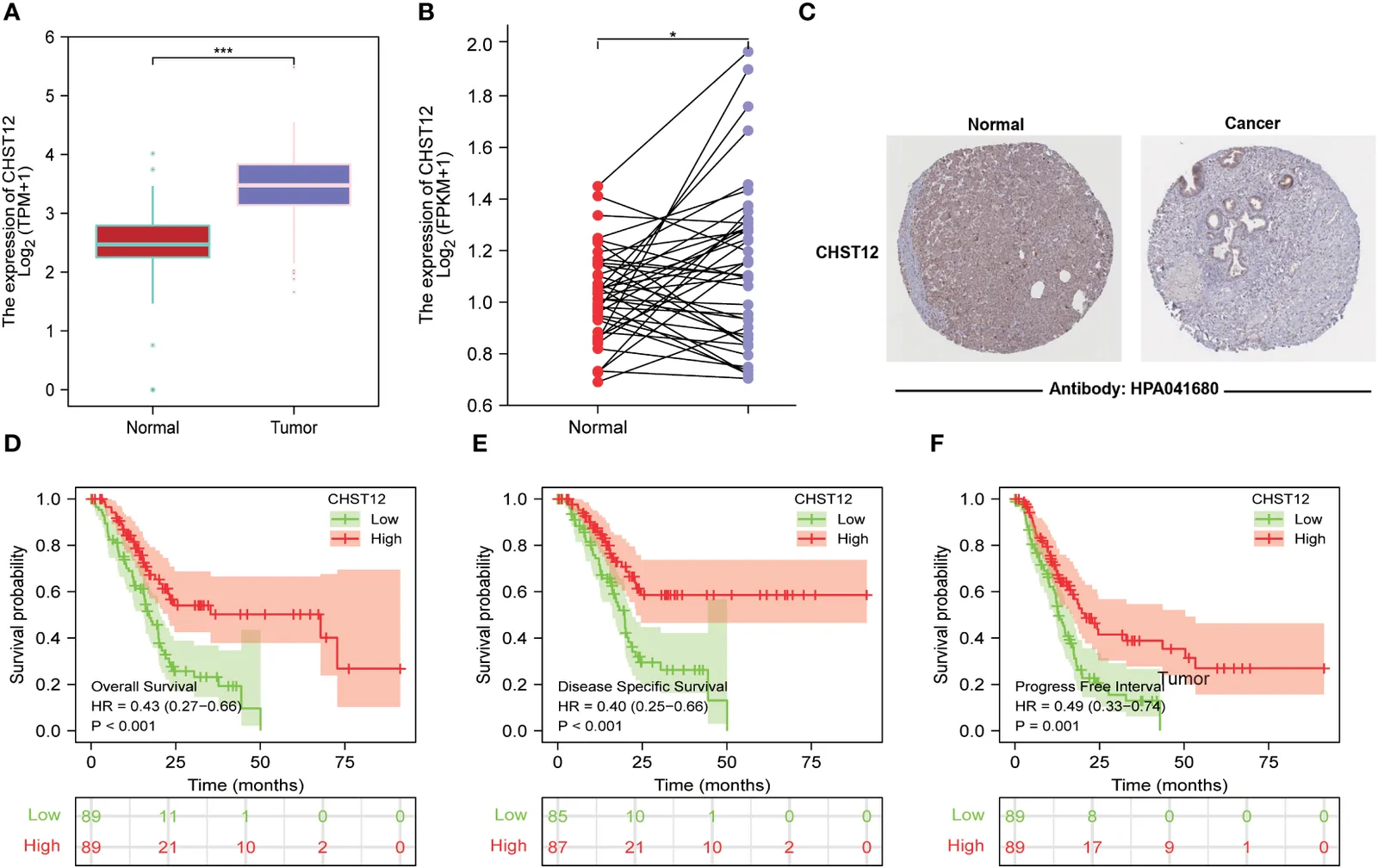
**(A)** Differential expression of CHST12 mRNA in normal and tumor tissues of patients with PAAD. The results show that CHST12 was highly expressed in pancreatic cancer tissues. **(B)** Differential expression of CHST12 mRNA in PAAD paired tumor and normal samples. The results show CHST12 was highly expressed in the PAAD paired tumor sample. **(C)** CHST12 proteins are differentially expressed in PAAD tumors and normal tissues according to THPA. The results show the opposite trend of mRNA levels, with lower levels of expression observed in PAAD tissues. **(D)** Survival curves comparing low to high expression groups of CHST12 mRNA for PAAD patients. **(E)** DSS. **(F)** PFI. The results show that low expression patients had a significantly shorter OS (p < 0.001, HR: 0.43), DSS (p< 0.001, HR: 0.40), and PFI (p = 0.001, HR: 0.49) than the high expression group. “*”:P<0.05, “***”:P<0.001.

In the TCGA+GTEx datasets, compared with normal tissues (N=171), tumor tissues (N=179, p < 0.001) expressed high levels of CHST12 mRNA. Likewise, in the paired analysis of normal and tumor tissues, CHST12 was highly expressed in pancreatic cancer tissues (N=41; p=0.038) (**Figures 1A, B**). We further analyzed the protein expression of CHST12 by immunohistochemistry. Interestingly, CHST12 protein levels from The Human Protein Atlas (THPA) database showed the opposite trend of mRNA levels, with lower levels of expression observed in PAAD tissues (**Figure 1C**). These results indicate an important post-transcriptional modification of CHST12.

To better explore the prognostic value of CHST12, patients from the TCGA dataset were initially classified into high and low groups based on the CHST12 mRNA level. Kaplan–Meier curves showed that low expression patients had a significantly shorter OS (p < 0.001; HR, 0.43), DSS (p < 0.001; HR, 0.40), and PFI (p = 0.001; HR, 0.49) than the high expression group (**Figures 1D–F**). These data suggest that CHST12 is a potential clinical outcomes-protected factor.

### 3.2 Correlation between CHST12 expression and the clinicopathological characteristics of PAAD

Our study examined the clinicopathological characteristics of patients with PAAD who expressed high and low CHST12 mRNA expression, as shown in **Table 1**. In terms of the distribution of clinicopathological characteristics, there was no significant difference (gender, age, T-stage, N-stage, M-stage, pathologic stage, radiation therapy, and primary therapy outcome). Furthermore, by using logistics analysis, we determined the correlation between CHST12 expression and clinicopathological characteristics (T stage, N stage, M stage, pathologic stage, primary therapy outcome, gender, age, histologic grade, anatomic neoplasm subdivision, alcohol history, history of diabetes, and family history of cancer). Prominently positive correlations between CHST12 expression and anatomic neoplasm subdivision and alcohol history are presented in **Table 2**.

**Table2:** Logistic regression analysis of the association between CHST12 expression and the clinicopathological characteristics in PAAD patients.

Characteristic	Total (N)	Odds ratio (OR)	P-value
T stage (T3&T4 vs. T1&T2)	176	0.925 (0.422-2.016)	0.843
N stage (N1 vs. N0)	173	1.032 (0.534-1.999)	0.926
M stage (M1 vs. M0)	84	1.538 (0.242-12.160)	0.647
Pathologic stage (stage III & stage IV vs. stage I & stage II)	175	1.012 (0.232-4.408)	0.987
Primary therapy outcome (PD & SD vs. CR &P R)	139	0.875 (0.443-1.720)	0.699
Gender (female vs. male)	178	1.095 (0.606-1.981)	0.763
Age (<=65 vs. >65)	178	1.371 (0.761-2.483)	0.294
Histologic grade (G3 & G4 vs. G1 & G2)	176	1.703 (0.881-3.349)	0.117
Anatomic neoplasm subdivision (other vs. head of pancreas)	178	2.211 (1.078-4.693)	**0.033**
Alcohol history (yes vs. no)	166	1.991 (1.061-3.791)	0.034
History of diabetes (yes vs. no)	146	1.375 (0.655-2.936)	0.403
Family history of cancer (yes vs. no)	110	1.173 (0.548-2.516)	0.680

Statistical significance was found between CHST12 expression and Anatomic neoplasm subdivision (other vs. head of pancreas) (P<0.05).

### 3.3 Diagnostic and prognostic predictive value of CHST12 in PAAD

**Figure 2:**
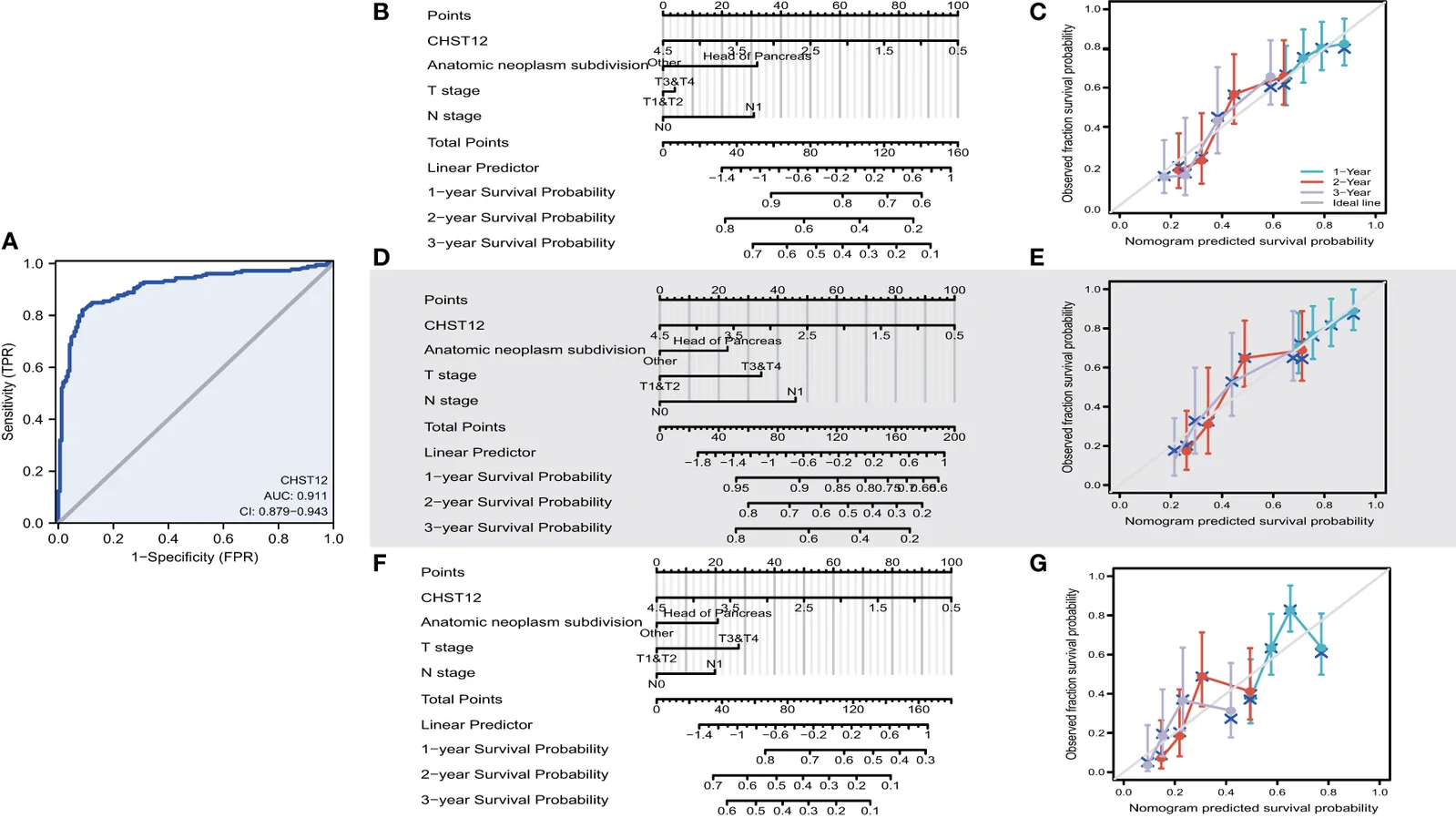
**(A)** ROC curve analysis for CHST12 clinical predictive values in PAAD patients. As the AUC was 0.911 (95% CI = 0.879–0.943), CHST12 showed high diagnostic efficacy, with 82.1% sensitivity and 91.2% specificity and a high positive predictive value (PPV) (90.7%) and negative predictive value (NPV) (83.0%) for the diagnosis of PAAD. **(B)** Nomograms of overall survival for CHSE12 expression-based risk scoring models predicting 1-, 2-, and 3-year clinical outcomes. **(C)** Calibration plots for the nomograms for overall survival. **(D)** Nomograms of the progress free interval (PFI) for CHSE12 expression-based risk scoring models predicting 1-, 2-, and 3-year clinical outcomes. **(E)** Calibration plots for the nomograms for PFI. **(F)** Nomograms of disease-specific survival (DSS) for CHSE12 expression-based risk scoring models predicting 1-, 2-, and 3-year clinical outcomes. **(G)** Calibration plots for the nomograms for DSS.

To investigate the predictive value for PAAD diagnosis, ROC curves and the AUC of CHST12 were calculated in PAAD to demonstrate its discriminating value. As the AUC was 0.911 (95% CI = 0.879–0.943), CHST12 showed high diagnostic efficacy, with 82.1% sensitivity and 91.2% specificity and a high positive predictive value (PPV) (90.7%) and negative predictive value (NPV) (83.0%) for the diagnosis of PAAD (**Figure 2A**). Next, we performed univariate and multivariate Cox regression analyses to identify the potential prognostic value of CHST12 for clinical outcomes. CHST12 mRNA expression was shown to be an independent risk factor for OS in a multivariate Cox regression analysis (HR, 0.617; P = 0.040) (**Table 3**). Similarly, the clinical N stage displayed a meaningful predictive value for DSS in PAAD. Here, key prognostic factors in OS, DSS, and PFI were selected as parameters for constructing nomograms to present the prognostic status of PAAD patients (**Figures 2B, D, F**). Then, to test the efficacy of the nomograms, calibration curves were drawn for the 1-, 2-, and 3-year clinical outcomes. A C-index of 0.619 (0.585-0.654) was obtained for CHST12 in the nomogram to predict OS (**Figure 2C**), DSS (**Figure 2E**), and PFI (**Figure 2G**).

**Table 3:** Cox regression analysis for clinical outcomes in PAAD patients.

Characteristic	Total (N)	HR for overall survival (95% CI)		HR for disease-specific survival (95% CI)		HR for progression-free intervals (95% CI)	
		Univariate	Multivariate	Univariate	Multivariate	Univariate	Multivariate
T stage	176						
T1&T2	31						
T3&T4	145	2.023 *	1.079	3.119 **	1.780	2.414 **	1.614
N stage	173						
N0	50						
N1	123	2.154 **	1.814*	2.746 **	2.162*	1.735 *	1.405
CHST12	178	0.478 ***	0.617*	0.508 *	0.658	0.505**	0.650
Anatomic neoplasm subdivision	178						
Head of pancreas	138						
Other	40	0.417 **	0.539	0.447 *	0.681	0.495 **	0.700

HR, hazard ratio; PAAD, pancreatic adenocarcinoma; CI, confidence interval. *P < 0.05; **P < 0.01; ***P < 0.001.

### 3.4 Functional annotation and identified CHST12-related signaling pathways in PAAD

**Figure 3:**
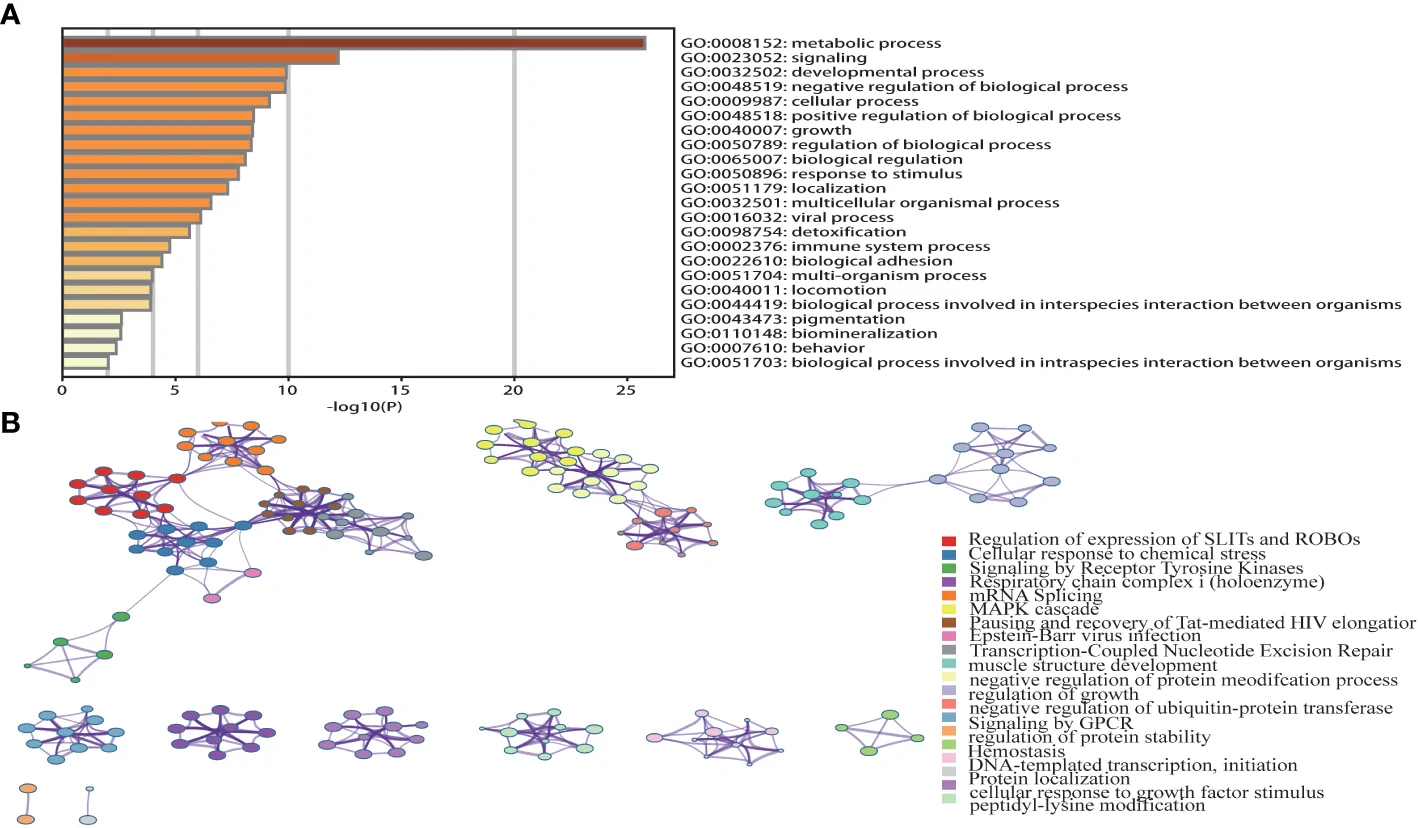
**(A)** Top 23 pathway clusters associated with CHST12-related DEGs enriched by Metascape. **(B)** TGraph of the top 20 enriched clustered terms. The term colors indicate nodes in close proximity to each other. An edge links terms that have a similarity score of >0.3 (the edge thickness corresponds to the similarity score). The darker the color, the greater the statistical significance. In the enrichment analysis, we found that the DEGs usually targeted tumor progression, immune-related signaling pathways, or biological processes that are usually involved in the tumor microenvironment.

A pathway enrichment analysis of CHST12-associated DEGs was conducted using the Metascape database, GO Biological Processes, KEGG Pathway, and Reactome Gene Set. These can help elucidate the functional role of CHST12-associated DEGs in PAAD patients with the criteria of false discovery rate (FDR) < 0.05 and |log2FC| > 1 (36). In the enrichment analysis, we found that the DEGs usually target tumor progression, immune-related signaling pathways, or biological processes that are usually involved in the tumor microenvironment. These may include immune system process, cellular response to growth factor stimulus, and signaling by receptor tyrosine kinases. (**Figures 3A, B**).

### 3.5 CHST12 may have a certain correlation with the immune infiltration of TME in PAAD

**Figure 4:**
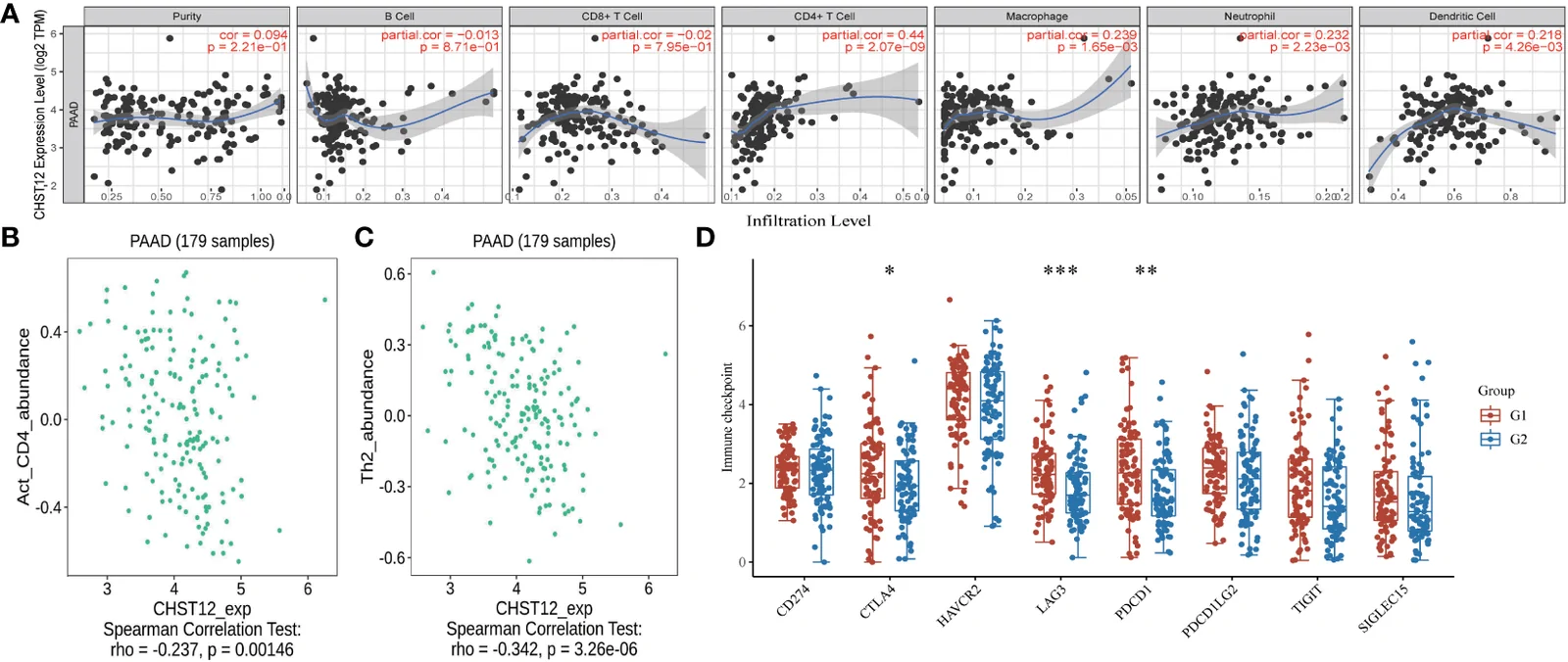
**(A)** A positive correlation was found between CHST12 expression and the infiltrating levels of T cells, macrophages, neutrophils, and dendritic cells; however, there was a negative correlation with CD8+ cells. **(B)** Correlation between CHST12 expression and the abundance of activated CD4 T cells in PAAD. **(C)** Correlation between CHST12 expression and the abundance of activated Th2 cells in PAAD. **(D)** CHST12 mRNA expression was associated with several immune checkpoint distributions, including PD-1, CTLA-4, and LAG-3 in PAAD.

A crucial role for glycosylation in modulating tumor immunity has been demonstrated and CHST12-associated DEG pathway analysis suggested that as a member of glycosylation, CHST12 may play a significant role in tumor microenvironment immunity in PAAD tumors. Hence, we tested whether there are correlations between CHST12 expression and TILs. We detected that higher CHST12 mRNA expression was associated with the increased infiltration of CD4+ T cells, macrophages, neutrophils, and dendritic cells. (**Figure 4A**). Using TISIDB analysis, our findings revealed that CHST12 mRNA was remarkably negatively correlated with CD4 and Type2 T-helper (Th2) cells, the infiltration of which was critical for immunotherapy producing anti-cancer immunity (**Figures 4B, C**). We further analyzed the relationship between CHST12 expression and the expression of immune checkpoints (CD274, CTLA4, HAVCR2, LAG3, PD-1 (PDCD1), PDCD1LG2, TIGIT, and SIGLEC15). Additionally, we proved that CHST12 mRNA expression was associated with several immune checkpoint distributions, including PD-1, CTLA-4, and LAG-3 in PAAD (**Figure 4D**). In summary, the expression pattern of CHST12 is closely related to immune cell infiltration in the TME of PAAD.

### 3.6 CHST12 contributes to immune escape and resistance to ICIs

**Figure 5:**
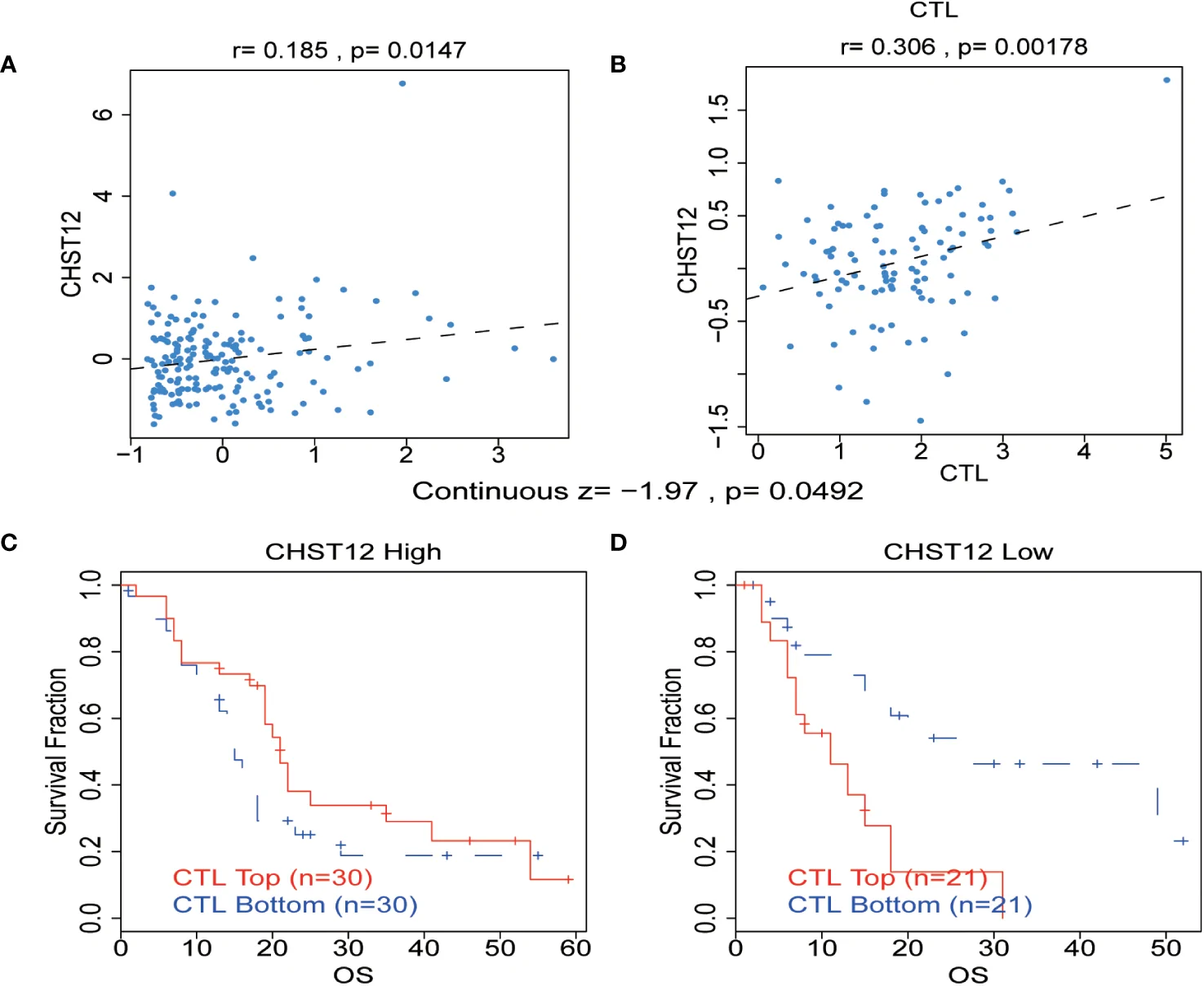
**(A, B)** Correlation between the levels of CHST12 and the CTL in the TME of PAAD from TCGA and GSE21501@PRECOG datasets. TCGA-PAAD: r = 0.185, p = 0.0147; GSE21501: r=0.306, p=0.00178) **(C, D)** The association between the CTL level and OS for PAAD with CHST12 high and low expression groups.

There is growing evidence that the tumor-infiltrating immune cells play a critical role in the TME and that higher levels of immune cell infiltration are associated with a better prognosis for tumor patients. Pearson correlation analysis using the TIDE web tool showed that increased CHST12 expression was associated with an increase in the number of cytotoxic T lymphocytes in two PAAD datasets (TCGA-PAAD and GSE21501@PRECOG, TCGA-PAAD; r = 0.185, p = 0.0147; GSE21501, r = 0.306, p = 0.00178) (**Figures 5A, B**). Moreover, we found that in the high CHST12 expression level group, high-level cytotoxic T lymphocyte (CTL) infiltration indicates better survival (**Figure 5C**); vice versa, in the low CHST12 expression level group, low CTL infiltration indicates better survival (**Figure 5D**). In the TIDE gene set prioritization module, tumor immune evasion phenotypes were not detected with a negative TIDE score. Furthermore, we examined the correlation between the CHST12 expression level and the efficacy of immunotherapy. Using the gene set prioritization module, we found that low CHST12 was related to an increase in the clinical efficacy of immunotherapy in melanoma and bladder cancer. These results suggest that CHST12 may be a potential candidate predictor for immunotherapy, especially ICIs.

## 4 Discussion

Carbohydrate sulfotransferases are enzymes that transfer sulfate to carbohydrate groups in glycoproteins, which have been implicated in the metastatic spread of tumor cells through the modulation of the TME (42–45). The deregulated expression of CHST family members has been reported in a wide range of human cancer types (33). Here, according to our findings, CHST12 mRNA expression was upregulated in PAAD, and low CHST12 expression predicted a favorable outcome for PAAD patients.

Through transcriptome analysis, we found that CHST12 mRNA was highly expressed in tumors, but CHST12 protein showed lower expression in PAAD tumor tissues than in normal tissues. The contradiction between the upregulation of CHST12 mRNA and downregulation of the protein may involve potential post-transcriptional modification mechanisms, such as RNA splicing or protein degradation. This result highly suggests that CHST12 has experienced post-transcriptional modification. Moreover, pancreatic cancer patients with low CHST12 protein expression have a worse prognosis, which is consistent with the findings of Han Nie et al. (46). Through multivariate regression analysis, we identified CHST12 and N staging as independent prognostic risk factors for OS in PAAD patients, which indicates that CHST12 may be useful in clinical settings as a prognostic biomarker and possesses potential clinical utility in PAAD patients. The ROC curve shows the diagnostic performance of CHST12 in discriminating PAAD diagnosis with an AUC of 0.911, suggesting that CHST12 is a high-performance biomarker for PAAD diagnosis. These results agree with previously reported results that other tumor patients with high CHST12 mRNA levels have shorter survival times (47, 48). Considering the key value of CHST12 in clinical diagnosis, we further investigated the potential functions and molecular mechanisms of CHST12 in PAAD. In our study, functional enrichment analysis of CHST12-associated DEGs showed that they are significantly enriched in tumor progression and immune-related signaling pathways. CHST12 has shown a similar effect in hepatocellular carcinoma (49). All the above analyses indicate that CHST12 may be involved in the regulation of the TME in PAAD.

Glycosylation plays a significant role in immunity and endows unique properties to glycoproteins (50, 51). Moreover, our functional studies have found that CHST12 plays a vital role in the TME. Hence, the effects of CHST12 expression on the TME and immunotherapy of pancreatic cancer is a key aspect of our study. According to our findings, CHST12 expression is positively correlated with the infiltration of immune cells (CD4+ T cells, macrophages, neutrophils, and dendritic cells), which contributes to immunosurveillance, the elimination of tumor cells, and the slowing of immune evasion in the PAAD microenvironment (52, 53). Additionally, we proved that CHST12 mRNA expression was associated with several immune checkpoint distributions, including PD-1, CTLA-4, and LAG-3, in PAAD. Using the gene set prioritization module, we found that low CHST12 was related to the increase in the clinical efficacy of immunotherapy in melanoma and bladder cancer. These results suggest that CHST12 may be a potential candidate predictor for immunotherapy, especially ICIs. To test the prediction, we will next conduct a verification experiment clinicopathologically. Combined with the results of the previous discovery of the post-transcriptional modification of CHST12, our study supports the notion that the decreased expression of CHST12 protein in the TME may produce an anti-tumor effect by acting on the recruitment of immune cells to the TME. Notably, in terms of CTLA4, LAG3, and PDCD1, the low expression CHST12 group differed significantly from the high expression CHST12 group. CTLA4, LAG3, and PDCD1 are important targets for cancer ICI therapy. This finding prompted us to investigate whether CHST12 expression impacts clinical outcomes when treated with ICIs. Using the TIDE algorithm, we predicted the response of patients to immunotherapy. We found that high CHST12 mRNA expression is related to the increased efficacy of immunotherapy in melanoma and bladder cancer. These data imply that CHST12 may be a predictor for the efficacy of immunotherapy.

Our findings uncover CHST12 as a new independent predictor of PAAD prognosis and immunotherapeutic efficacy, but it has its limitations. First, CHST12 expression and its prognostic significance were identified through bioinformatic analysis. Hence, further study with clinical samples for such applications is needed to verify the above findings. In addition, *in vivo* and *in vitro* experiments are needed to provide additional information about how CHST12 impacts immune infiltration in the PAAD TME.

Conclusively, in this study, we have shown that CHST12 mRNA expression is upregulated in PAAD, whereas CHST12 protein expression is downregulated. The expression of CHST12 is an independent predictor of a good prognosis in PAAD. In addition, CHST12 may affect the infiltration of immune cells in the microenvironment of PAAD. Our data highlight the potential of CHST12 in predicting ICI efficacy. In the future, we hope to further investigate the regulatory mechanisms of CHST12 through animal trials to test its validation. Additionally, we hope to conduct clinical trials as well. Overall, CHST12 may be a novel prognosis biomarker and a potential predictor of the response to ICI treatment in PAAD patients.

## Data Availability

The data sets used and/or analyzed during the current study arec available from the following public data sources: the TCGA-PAAD (https://xenabrowser.net/datapages/) and GSE21501 (https://ncbi.nlm.nih.gov/geo/query/acc.cgi?acc=GSE21501) data sets. Publicly available datasets were analyzed in this study. These data can be obtained from the following sites: http://www.xiantao.love; https://www.proteinatlas.org/; https://metascape.org/; http://tide.dfci.harvard.edu/; http://cis.hku.hk/TISIDB/.
