# Correction: CHST12: a potential prognostic biomarker related to the immunotherapy response in pancreatic adenocarcinoma

**DOI:** 10.3389/fendo.2026.1975559

**Published:** 2026-10-28

**Authors:** Kun Liu, Lu Li, Guang Han

**Affiliations:** Department of Anesthesiology, Shengjing Hospital of China Medical University, Shenyang, Liaoning, China

**Keywords:** immune cell, infiltration, glycosylation (enzymatic conjugation with carbohydrates), prognostic signature, pancreatic adenocarcinoma (PAAD), tumor microenvironment

Reference 1 was erroneously written as “Li C, Lin L, Zhang L, Xu R, Chen X, Ji J, et al. Long noncoding RNA p21 enhances autophagy to alleviate endothelial progenitor cells damage and promote endothelial repair in hypertension through SESN2/AMPK/TSC2 pathway. Pharmacol Res (2021)173:105920. doi: 10.1016/j.phrs.2021.105920.” It should be “Gavaghan M. The pancreas--hermit of the abdomen. Aorn j. 2002;75(6):1110-4, 7, 9 passim; quiz 31-2, 34-5, 37-8. doi: 10.1016/s0001-2092(06)61613-x.”

Reference 2 was erroneously written as “Zhu Y, Huang R, Wu Z, Song S, Cheng L, Zhu R. Deep learning-based predictive identification of neural stem cell differentiation. Nat Commun (2021) 12(1):2614. doi: 10.1038/s41467-021-22758-0.” It should be removed entirely.

Reference 3 was erroneously written as “Han S, Chen C, Chen C, Wu L, Wu X, Lu C, et al. Coupling annealed silver nanoparticles with a porous silicon Bragg mirror SERS substrate and machine learning for rapid non-invasive disease diagnosis. Analytica Chimica Acta (2023) 1254:341116. doi: 10.1016/j.aca.2023.341116.” It should be “Röder PV, Wu B, Liu Y, Han W. Pancreatic regulation of glucose homeostasis. Exp Mol Med. 2016;48(3):e219. doi: 10.1038/emm.2016.6.”

Reference 4 was erroneously written as “Sun Q, Tao Q, Ming T, Tang S, Zhao H, Liu M, et al. Berberine is a suppressor of Hedgehog signaling cascade in colorectal cancer. Phytomedicine (2023) 114:154792. doi: 10.1016/j.phymed.2023.154792.” It should be “Czakó L, Hegyi P, Rakonczay Z, Jr., Wittmann T, Otsuki M. Interactions between the endocrine and exocrine pancreas and their clinical relevance. Pancreatology. 2009;9(4):351-9. doi: 10.1159/000181169.”

Reference 5 was erroneously written as “Siegel RL, Miller KD, Jemal A. Cancer statistics, 2020. CA Cancer J Clin (2020) 70(1):7–30. doi: 10.3322/caac.21590.” It should be “Kleeff J, Korc M, Apte M, La Vecchia C, Johnson CD, Biankin AV, et al. Pancreatic cancer. Nat Rev Dis Primers. 2016;2:16022. doi: 10.1038/nrdp.2016.22.”

Reference 7 was erroneously written as “Vogelstein B, Papadopoulos N, Velculescu VE, Zhou S, Diaz LAJr, Kinzler KW. Cancer genome landscapes. Science (2013) 339(6127):1546–58. doi: 10.1126/science.1235122.” It should be “Beatty GL, Gladney WL. Immune escape mechanisms as a guide for cancer immunotherapy. Clin Cancer Res. 2015;21(4):687-92. doi: 10.1158/1078-0432.Ccr-14-1860.”

Reference 9 was erroneously written as “Li Q, Miao Y, Zeng X, Tarimo CS, WuC, WuJ.Prevalence and factors for anxiety during the coronavirus disease 2019 (COVID-19) epidemic among the teachers in China. J Affect Disord (2020) 277:153–8. doi: 10.1016/j.jad.2020.08.017.” It should be “Vincent A, Herman J, Schulick R, Hruban RH, Goggins M. Pancreatic cancer. Lancet. 2011;378(9791):607-20. doi: 10.1016/s0140-6736(10)62307-0.”

Reference 10 was erroneously written as “Xu H, van der Jeught K, Zhou Z, Zhang L, Yu T, Sun Y, et al. Atractylenolide I enhances responsiveness to immune checkpoint blockade therapy by activating tumor antigen presentation. J Clin Invest (2021) 131(10):e146832. doi: 10.1172/JCI146832.” It should be “McGuigan A, Kelly P, Turkington RC, Jones C, Coleman HG, McCain RS. Pancreatic cancer: A review of clinical diagnosis, epidemiology, treatment and outcomes. World J Gastroenterol. 2018;24(43):4846-61. doi: 10.3748/wjg.v24.i43.4846.”

Reference 11 was erroneously written as “Abbas M, Alqahtani MS, Alshahrani MY, Alabdullh K. Aggressive and drug resistant pancreatic cancer: challenges and novel treatment approaches. Discovery Med (2022) 34(173):158–64.” It should be “Kunz PL, Fisher GA. Advances in the treatment of gastroenteropancreatic neuroendocrine tumors. Clin Exp Gastroenterol. 2010;3:79-86. doi: 10.2147/ceg.s5928.”

Reference 12 was erroneously written as “Liu G, Li B, Xu Z, Wang J, Ma S, Kan Y, et al. Bacillus calmette-guerin for the treatment of non-muscle invasive bladder cancer: history and current status. Discovery Med (2022) 33(169):85–92.” It should be “Yao JC, Lombard-Bohas C, Baudin E, Kvols LK, Rougier P, Ruszniewski P, et al. Daily oral everolimus activity in patients with metastatic pancreatic neuroendocrine tumors after failure of cytotoxic chemotherapy: a phase II trial. J Clin Oncol. 2010;28(1):69-76. doi: 10.1200/jco.2009.24.2669.”

Reference 13 was erroneously written as “Hui L, Chen Y. Tumor microenvironment: Sanctuary of the devil. Cancer Lett (2015) 368(1):7–13. doi: 10.1016/j.canlet.2015.07.039.” It should be removed entirely.

Reference 18 was erroneously written as “OhD,Seo DW,SongTJ, Park DH, Lee SK, Kim MH. Clinical outcomes of EUS-guided radiofrequency ablation for unresectable pancreatic cancer: A prospective observational study. Endosc Ultrasound Jan-Feb (2022) 11(1):68–74. doi: 10.4103/eus-d-21-00049.” It should be “Krishna SG, Brugge WR, Dewitt JM, Kongkam P, Napoleon B, Robles-Medranda C, et al. Needle-based confocal laser endomicroscopy for the diagnosis of pancreatic cystic lesions: an international external interobserver and intraobserver study (with videos). Gastrointest Endosc. 2017;86(4):644-54.e2. doi: 10.1016/j.gie.2017.03.002.”

Reference 20 was erroneously written as “Nakai A, Kamata K, Hyodo T, Chikugo T, Hara A, Otsuka Y, et al. Utility of contrast-enhanced harmonic EUS for diagnosis of portal vein invasion by pancreatic cancer. Endosc Ultrasound (2022) 11(5):401–6. doi: 10.4103/eus-d-21-00185.” It should be removed entirely.

Reference 27 was erroneously written as “Zhang Z, Wang L, Zheng W, Yin L, Hu R, Yang B. Endoscope image mosaic based on pyramid ORB. Biomed Signal Process control (2022) 71:103261. doi: 10.1016/j.bspc.2021.103261.” It should be “Silva ML. Cancer serum biomarkers based on aberrant post-translational modifications of glycoproteins: Clinical value and discovery strategies. Biochim Biophys Acta. 2015;1856(2):165-77. doi: 10.1016/j.bbcan.2015.07.002.”

Reference 38 was erroneously written as “Lu S, Yang B, Xiao Y, Liu S, Liu M, Yin L, et al. Iterative reconstruction of low dose CT based on differential sparse. Biomed Signal Process Control (2023) 79:104204. doi: 10.1016/j.bspc.2022.104204.” It should be removed entirely.

Reference 51 was erroneously written as “Matsumoto H, Minaga K, Nakano S, Uenoyama Y. Very late distant retroperitoneal metastasis of breast cancer diagnosed using EUS-guided tissue acquisition. Endosc Ultrasound (2022) 12(2):279–80. doi: 10.4103/eus-d-22-00050.” It should be “Zhang C, Wang M, Ji F, Peng Y, Wang B, Zhao J, et al. A Novel Glucose Metabolism-Related Gene Signature for Overall Survival Prediction in Patients with Glioblastoma. Biomed Res Int. 2021;2021:8872977. doi: 10.1155/2021/8872977.”

Reference 52 was erroneously written as “Zhang L, Fan Y, Wang X, Yang M, Wu X, Huang W, et al. Carbohydrate sulfotransferase 4 inhibits the progression of hepatitis B virus-related hepatocellular carcinoma and is a potential prognostic marker in several tumors. Front Oncol (2020) 10:554331. doi: 10.3389/fonc.2020.554331.” It should be “Cheng S, Guo J, Zhang Y, Li Z, Li C. Identification of a multidimensional transcriptome signature predicting tumor regrowth of clinically non-functioning pituitary adenoma. Int J Oncol. 2020;57(3):804-12. doi: 10.3892/ijo.2020.5087.”

The original version of this article has been updated.

